# Interlayer Atomic Voids by Partial Cesium Defect in Layered Titanate Activate Photo(electro)catalytic H_2_ and O_2_ Generation

**DOI:** 10.1021/acsaem.5c01514

**Published:** 2025-09-11

**Authors:** Tuğçe Üstünel, José Julio Gutiérrez Moreno, Xiaoran Zheng, Sajjad S. Mofarah, Hadi Jahangiri, Sarp Kaya, Esmail Doustkhah

**Affiliations:** † Materials Science and Engineering, 52979 Koç University, Istanbul 34450, Türkiye; ‡ Koç University Hydrogen Technologies Center (KUHyTech), Istanbul 34450, Türkiye; § 132144 Barcelona Supercomputing Center, Plaça d’Eusebi Güell, 1-3, Barcelona 08034, Spain; ∥ School of Materials Science and Engineering, 7800 UNSW Sydney, Sydney, New South Wales 2052, Australia; ⊥ Koç University Surface Science and Technology Center (KUYTAM), Koç University, Sariyer, Istanbul 34450, Türkiye; # Department of Chemistry, Koç University, Istanbul 34450, Türkiye; ¶ Chemistry Department, Faculty of Engineering and Natural Sciences, 469683 Istinye University, Sariyer, Istanbul 34396, Türkiye; ∇ Clean Energy Research Center (TEAM), Istinye University, Sariyer, Istanbul 34396, Türkiye

**Keywords:** band gap engineering, layered semiconductors, hydrogen generation, photoelectrocatalysis, cation vacancy

## Abstract

Although many layered oxide semiconductors possess seemingly suitable band gaps for photo­(electro)­catalysis in the UV–vis range, they often exhibit low or no activity in practice. Bulk layered cesium titanate is one such example of an inactive semiconductor, where the introduction of cesium vacancies with subsequent thermal treatment leads to the activation of its photocatalytic properties. Here, we demonstrate the promising effect of cesium vacancies on the photocatalytic (PC) activity enhancement in the hydrogen evolution reaction (HER) from water in the presence of H_2_O/MeOH (80:20). In a separate experiment, we also further prove that the Cs-vacant (V_Cs_) layered titanate sample treated at 700 °C exhibits a remarkably improved photoelectrocatalytic (PEC) activity in the oxygen evolution reaction (OER) of partially exfoliated cesium titanates. In contrast, bulk cesium titanate shows no PC activity toward HER and only minimal PEC activity toward OER. Computational modeling reveals that partial interlayer Cs vacancies can increase the surface area up to 120 Å^2^ and generate interlayer voids as large as 10 Å. Furthermore, hybrid density functional theory (DFT) calculations indicate that these Cs vacancy defects lead to the formation of midgap states, which are expected to enhance photogenerated charge carrier separation and stabilization, thereby improving both PC and PEC activities. Our approach results in the development of a cocatalyst-free semiconductor light absorber capable of producing hydrogen with significantly higher efficiency than both bulk cesium titanate and protonated layered titanate.

## Graphical abstract

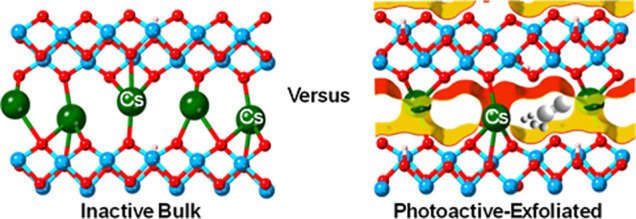

## Introduction

Photoelectrocatalytic (PEC) and photocatalytic (PC) conversion processes can play key roles in contributing to a neutral carbon footprint by utilizing sunlight in the water splitting processes, such as oxygen evolution (OER) and hydrogen evolution reactions (HER).^1–4^ Solar light harvesting on photoelectrodes as a free energy source can vastly synergize the catalysis, leading to a reduction in the process cost.^3,5,6^ However, the genuine industrialization of PC and PEC processes has some hurdles.^1^ OER, with its high redox potential value (1.23 eV), is the main reason why water splitting is a costly process.^7–9^ On the other hand, the efficiency of the solar-to-hydrogen on a semiconductor can be constrained by the large band gap energy (*E*_g_), fast electron–hole recombination, and poor surface activities toward HER and OER.^10–12^ Even though some of the semiconductors have proper band gaps and band edge potential levels, few of them show promising activity in the PC/PEC water splitting.^13^ Therefore, semiconductors are required to be investigated in depth to understand their PC/PEC mechanisms to evolve them toward state-of-the-art activity and stability by understanding how they should be subsequently modified.

An example of such photocatalysts for water splitting is titanium-based oxides, usually accompanied by cocatalysts and/or post-modifications. This is due to their advantageous band edge positions, efficient light absorption, high stability in acidic and alkaline solutions, and low cost.^14^ In contrast, bulk layered titanates^15^ (i.e., cesium titanate) with a high cation exchange capability^16^ could be taken as examples of photocatalytically poor semiconductors that suffer from having large band gaps, low surface area, and low stability under acidic and thermal conditions, and therefore, low PC/PEC activities.^10,17^ Modifications of their interlayer chemistry,^15^ such as enhancing the surface area and quantum confinement properties through exfoliation, have been widely achieved through various methods.^18–20^ The particle size reduction^21^ and proton exchange^20^ are two other promising strategies to convert bulk layered titanate into a photocatalytically active layered nanomaterial that also leads to a red shift in the band gap and PC improvement.^20^ Treating layered titanates with alkylamine- or alkylammonium-based species to exfoliate the layers via a cation-exchange method represents another distinct approach to enhancing PC activity, primarily by increasing the surface area and reducing the band gap.^22–24^ The synthesis of a heterostructure composed of layered titanate and another material, either intercalated or supported, can further enhance the activity of the layered titanate; such a configuration can also be referred to as a heterojunction.^25–29^ Such modifications can enhance mass transfer and diffusion due to increased surface area, improve adsorption activity and charge separation, narrow the band gap, reduce charge carrier resistance, and ultimately boost PC and PEC performance.^17,30^ Like other oxides, defect engineering and doping in layered titanates can play a pivotal role in enhancing PC and PEC activity by reducing charge carrier resistance, introducing midgap states, improving catalytic sites, narrowing the band gap, and prolonging the lifetime of photogenerated electron–hole pairs.^19,31–35^

To achieve high PC and PEC performance in layered titanates, we utilized a cesium-based layered titanate (CsTO), where the atomic size of Cs is relatively larger than that of conventional alkali metals (e.g., Na, Li, or K cations), as well as the other elements of the structure (Ti, O, and H). Its partial removal from the interlayer space can introduce significant interatomic voids within the layers. This partial Cs vacancy was induced by simultaneous partial exfoliation of the layers through the treatment of bulk CsTO with alkyl ammonium species, followed by partial cation exchange.

## Experimental Procedures

### Materials and Characterization

All reagents and solvents were purchased commercially and used without further purification. TiO_2_ (P25; 97%), Cs_2_CO_3_ (99.9%), and cetyltrimethylammonium bromide (≥98%) were purchased from Sigma-Aldrich. For all the experiments, ultrapure water (≥18.2 MΩ·cm) was used. X-ray diffraction (XRD) measurements were conducted by using a powder X-ray diffractometer (Bruker D2 Phaser X-ray Diffractometer) with Cu Kα radiation, with a 10 kV beam voltage and a 30 mA current. Scanning electron microscopy (SEM) measurements were taken with a Zeiss Ultra Plus field emission scanning electron microscope with an accelerating potential of 20 kV by placing the samples on carbon tape. High-resolution transmission electron microscopy (HRTEM) images, selected area electron diffraction (SAED) patterns, and energy dispersive X-ray spectroscopy (EDS) of the nanostructures were obtained using a JEOL JEM-F200 S/TEM, operated at 200 kV. The samples were dispersed in ethanol and sonicated to drop-cast onto the shiny side of the holey carbon-coated Cu grid (200 mesh). The grids dried in a dust-free environment at room temperature overnight to ensure complete solvent evaporation. Then, it was transferred into the instrument for operating both TEM and STEM. The corresponding EDS spectra were collected and operated in both secondary and backscattered electron modes, with an accelerating voltage ranging from 10 to 15 kV. The microscope was fitted with a 100 mm^2^ silicon drift detector, providing high sensitivity for elemental mapping. UV–vis spectroscopy measurements were performed on the powder mode using a Shimadzu UV-3600 UV–vis–NIR spectrophotometer in the reflection mode. The Kubelka–Munk transformation was applied prior to their extrapolation into the Tauc plots. Raman spectroscopy measurements were done by using a Renishaw inVia Raman Microscope with a 633 nm excitation laser source. X-ray photoelectron spectroscopy (XPS) measurements were conducted using a Thermo K-alpha spectrometer system with Al Kα radiation (*h*υ = 1486.7 eV) as the excitation source. Photoluminescence (PL) emission and lifetime measurements were performed using an Edinburgh Instruments FLS1000 spectrometer equipped with a picosecond diode laser. For this measurement, the powder samples were first dispersed in a water–methanol mixture (50% v/v) and transferred into the PL cells for further measurement at room temperature by exciting the samples through a 377 nm pulsed light-emitting diodes (LEDs) laser for lifetime decay measurment and through a 290 nm wavelength for PL emission measurment. Fourier transform infrared (FTIR) measurements were performed by Thermofisher Scientific Nicolet iS10 FT-IR spectrometer with a resolution of 4 cm^–1^. Thermogravimetric analysis (TGA) was done by a TA Instruments Q500 thermogravimetric analyzer.

### Preparation of Cesium Titanate (Cs_2_Ti_6_O_13_)

Layered cesium titanate, Cs_2_Ti_6_O_13_ (denoted as CsTO), was prepared by the solid-state reaction method using a molar ratio of TiO_2_ and Cs_2_CO_3_, 3:1, respectively. Materials were ground in an agate mortar with a pestle for 1 h, and the powder mixture was calcined in a muffle furnace at 800 °C for 20 h.

### Synthesis of Cs-Vacant exf-CsTO

For the synthesis of Cs-vacant CsTO, it was first exchanged with cetyltrimethylammonium bromide (CTAB) to partially remove the Cs ions while it is being disaggregated and exfoliated. Accordingly, the as-prepared CsTO (1 g) was dispersed in the CTAB solution (0.25 M, 100 mL) for 3 days, then centrifuged (2000 rpm) and washed with distilled water (20 mL) three times. Then, the precipitate dried overnight in a vacuum oven at room temperature. The CTAB-exchanged CsTO (CTAB_0.25_-CsTO) was then thermally treated in N_2_ flow (50 mL/min) at 600 °C, 700 °C, and 800 °C for 5 h with the temperature ramp rate of 2 °C/min in a quartz tube for a homogeneous treatment. After the thermal treatment, samples were named with corresponding temperatures, such as exf-CsTO-*x* (where the *x* indicates the temperature of calcination, and *x* can be 600, 700, 800).

### Photoelectrocatalytic Performance Tests

For photoelectrochemical performance tests, 6 mg of catalyst was dispersed in 5 mL of methanol solution, sonicated for 20 min to make sure that all the catalyst is well dispersed. Then, the solution was coated on a preheated (50 °C) indium tin oxide (ITO) surface (1 cm × 1 cm) by the drop-casting method. The ITO substrates were cleaned in ethanol, acetone, and deionized water in a sonicator bath prior to use. The coated thin film samples were left on a heater at 50 °C to dry for 10 min. All the PEC tests were carried out by a Biologic VSP300 potentiostat (with an error limit of < ± 1 mV ± 0.03% of reading through the device) through a 3-electrode configuration setup by using Ag/AgCl as a reference electrode, Pt wire as a counter electrode, and CsTO-coated ITOs as the working electrode in a quartz cell under irradiation by a Xe lamp (300 W). Linear sweep voltammetry (LSV) measurements to determine PEC OER activities were performed under chopped light conditions in 0.1 M Na_2_SO_4_. The potential scanning rate was set to 10 mV/s during LSV scans. Electrochemical impedance spectroscopy (EIS) measurements were taken in the dark at a potential amplitude of 10 mV over a frequency range of 100 kHz to 0.1 Hz. The obtained data were plotted as Nyquist plots and then fitted with Randle’s circuit equivalent circuit model. The conversion of the measured potential values from *V*_Ag/AgCl_ to *V*_RHE_ (RHE: reversible hydrogen electrode) was performed using the Nernst equation: *V*_RHE_ = *V*_Ag/AgCl_ + 0.059 × pH + *V*_Ag/AgCl°_, where *V*_Ag/AgCl°_ is 0.197 V.

### Photocatalytic Performance Tests

PC HER tests from water were carried out by dispersing 20 mg of catalyst in 15 mL of distilled water (20% V/V methanol as a hole scavenger) in a Pyrex test tube with a water-circulating side-cooling jacket sealed with a rubber septum and directly connected to the GC through pneumatic plastic tube to prevent any leakage of hydrogen gas. The test tube was illuminated with a 300 W xenon lamp at a 10 cm distance while stirring constantly. The amount of hydrogen produced during the reaction was quantified by transferring the evolved gas directly to the GC. The produced hydrogen amount was determined by a gas chromatograph (7820A, GC-System, Agilent) equipped with a thermal conductivity detector (TCD) and flame ionization detector (FID) while He was utilized as carrier gas. The error limit for the detection of measuring hydrogen through the developed method on GC was at least ±1 μmol. All photo­(electro)­chemical experiments were performed via Newport Oriel, equipped with a xenon lamp light source and an AM1.5G air mass filter, which corresponds to 1 sun (100 W/cm^2^) irradiance output.

### Computational Methods

The structural and electronic properties of the layered compound were analyzed at the atomic level using ab initio density functional theory (DFT) calculations. Initial calculations were carried out with SIESTA 5.0,^36,37^ which provides accurate information on the structure of the compound, making it optimal for use in computational resources. All calculations used double-zeta polarized (DZP) basis sets, the PBEsol exchange–correlation functional^38^ and its corresponding pseudopotential obtained from the Pseudodojo repository.^39^ Long-range interactions were included using Grimme’s D3 correction.^40^ The electronic and force convergence were set to 10^–6^ eV and 0.02 eV/Å, respectively. The initial relaxation maintained fixed lattice parameters and symmetry while allowing for manual adjustment of the interlayer distance. The structural optimization was performed using a two-step approach. The initial relation was done on a Monkhorst–Pack *k*-point mesh of 2 × 6 × 2 and a mesh cutoff of 500 Ry. The total energy and lattice parameters were fitted using the Birch–Murnaghan equation of state. In the second phase of optimization, the lattice parameters were refined by allowing the unit cell to fully relax while maintaining symmetrical constraints. This refinement employed more stringent computational parameters with 4 × 12 × 4 *k*-points and an energy cutoff of 800 Ry.

The electronic features were extracted from plane-wave DFT calculations carried out with Quantum Espresso.^41–43^ This code allows the use of hybrid DFT/Hartree–Fock methods, crucial for describing electronic structure accurately at the band gap level. To diminish the possible effect of forces derived from the use of the different basis sets, the ions were initially relaxed using identical exchange–correlation, long-range correction, pseudopotentials, *k*-point grids, and convergence criteria. A kinetic energy cutoff of 65 Ry was set for wave functions and 520 Ry for charge density and potential. From the relaxed structure, single-point spin-polarized hybrid DFT calculations using the HSE functional,^44^ and a *k*-point grid of 2 × 6 × 2 were employed to extract the density of states (DOS).

Structural analyses at the interface level were carried out with the Zeo++ package,^45,46^ a code designed for geometry-based analysis of porous materials, that uses Voronoi decomposition to analyze the void spaces. For the surface area calculations, which provide the area accessible by the center of a spherical probe, we determined a diameter of 0.2 Å. After localizing the pores, we executed a Monte Carlo sampling procedure with 100,000 samples to integrate surface area. To consider only the interfacial area, the values obtained in the pristine TiO_2_ model have been subtracted from the output of the interface calculations. Figures of the DFT-relaxed structural models were produced with CrystalMaker software, DOS graphs were produced using the sumo plotting tool.^47^

## Results and Discussion

Here, we utilized a new approach to activate the PC and PEC performance of CsTO. We first partially exchanged Cs^+^ with C_16_TMA^+^, and meanwhile, exfoliated the stacked layers. The cation-exchange of the alkali metals of layered titanates with alkylammonium species is well-established research that has been previously developed.^15,25,48^ The alkylammonium chains are usually intercalated in the interlayers of layered titanates, but in our case, probably due to the large lateral sizes of the CsTO, the intercalation did not occur efficiently, but only cation exchange Cs^+^ with a concentrated alkylammonium chain (0.25 M) under aqueous conditions (judging from XRD in Figure 1a). Then, the sample was heat-treated to remove the organic parts and converted into a partially exfoliated Cs-vacant layered titanate structure, where the Cs-vacant space can leave interatomic voids, modify the band gap, and eventually lead to PC/PEC activity enhancement. The schematic synthesis pathway of the photocatalyst is represented in Scheme 1.

**Figure 1:**
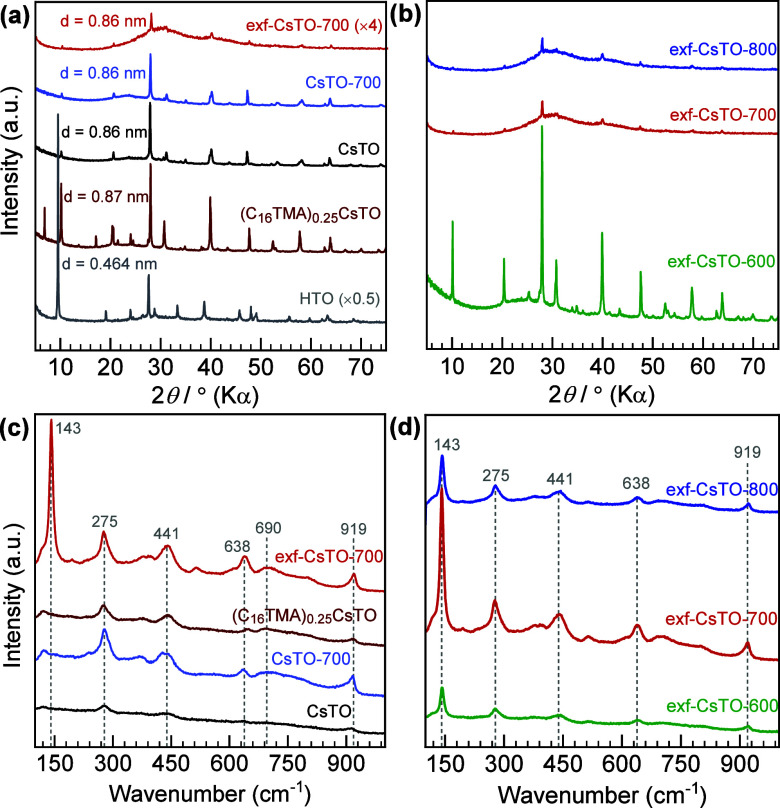
(a) XRD patterns of pristine CsTO, (C_16_TMA)_0.25_CsTO, exf-CsTO-700, CsTO-700, and HTO. (b) The heat-treated samples were at different temperatures, including 600 °C, 700 °C, and 800 °C. (c) Raman spectra of exf-CsTO-700 versus its pristine form and its unintercalated and heat-treated CsTO form. (d) The Raman spectra of the heat-treated CsTO (e.g., exf-exf-CsTO-600, exf-CsTO-700, exf-CsTO-800).

**Scheme 1:**
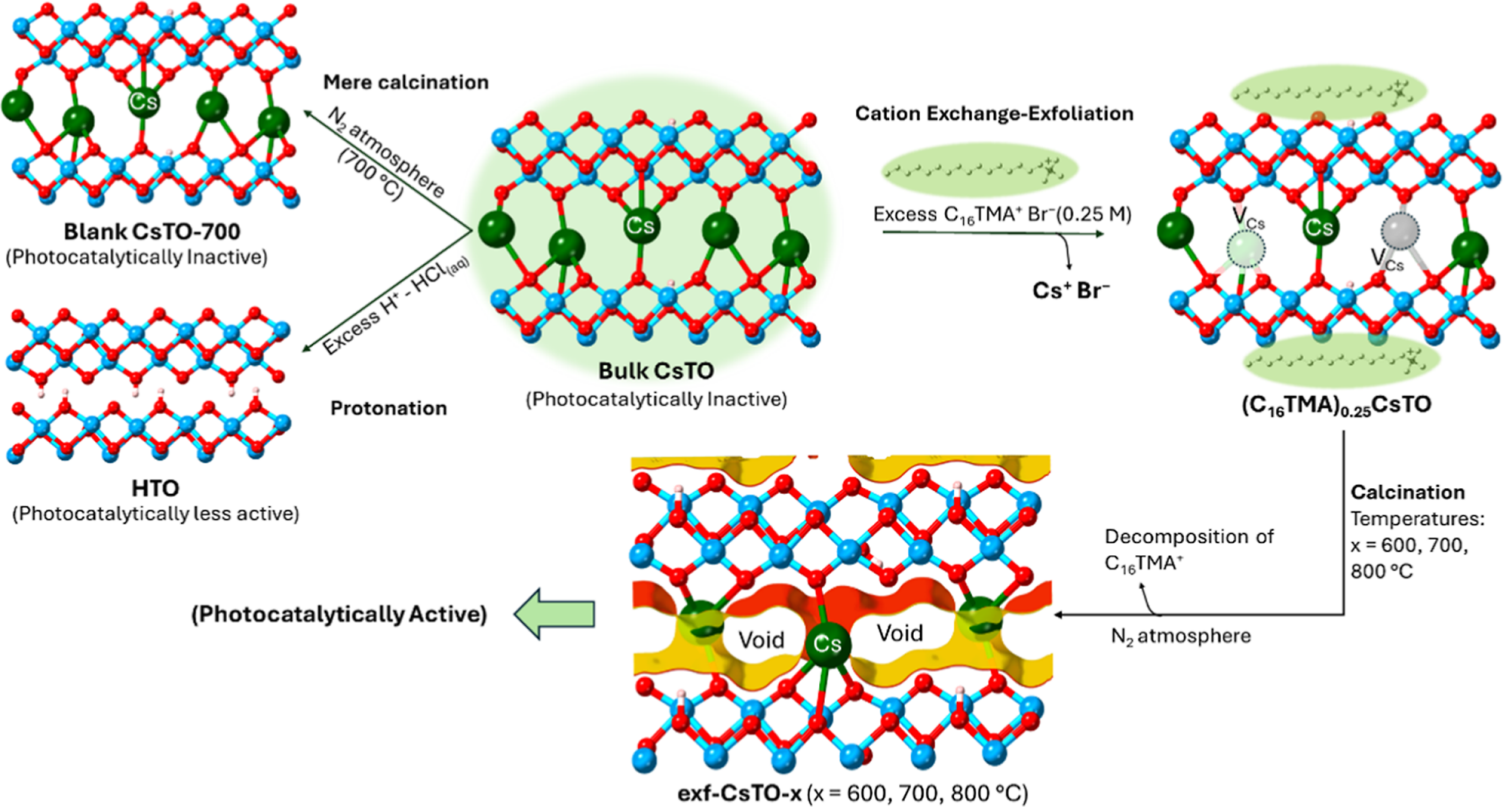
General Overview of the Interatomic Void Generation in the Structure of the Layered Titanate with the method described in this work^a^

a (Color coding for all figures in the paper: green for Cs, red for oxygen, blue for Ti, and while for H atoms). The blank CsTO-700 is when the bulk CsTO directly undergoes heat treatment at 700 °C without treating with C_16_TMA^+^.

The XRD patterns presented in Figure 1a illustrate that the synthesized CsTO has a structure corresponding to the PDF reference XRD pattern of Cs_2_Ti_6_O_13_, which consists of the orthorhombic phase (PDF card number: 00-038-0170, Figure S1). The obtained lattice parameters for the CsTO are also represented in Supporting Information, which is calculated from the XRD pattern of CsTO and assigned by the Miller indices of the represented Cs_2_Ti_6_O_13_ crystal structure in Table S1. The lattice parameters can vary based on the synthesis conditions, such as synthesis temperature, hydrated water molecules, and the time of heat treatment.^49^ The changes in the crystalline structure of the CsTO after treating with C_16_TMA^+^ are discernible by looking at the XRD pattern of (C_16_TMA)_0.25_CsTO. The first peak at 6.8° can be ascribed to the excessive remaining C_16_TMABr’s crystal structure (see Figure S1). The second peak at 10.1°, which is intensified (compared to the bulk CsTO), can be discernible to the unintercalated but significantly cation-exchanged interlayers.^50^ The fully protonated layered titanate (HTO) also reveals a peak at 9.6° that can be assigned to the interlayer d value about 0.9 nm, conveying that there is no bulky species in the interlayer space except proton, since the thickness of titanate is ∼0.8 nm.^51^

The *d* spacing value of HTO is larger than that of the (C_16_TMA)_0.25_CsTO, which can be due to the intercalation of water molecules in the case of HTO since the treatment was performed in acidic conditions. It is well established that the intercalation of cationic alkylammoniums in layered materials can expand the interlayer *d* spacing value, depending upon the length of the alkylammonium chain.^48,52^ However, herein, we did not observe any evidence of the intercalation, which could be hindered at the microlateral size of CsTO. More details on the structural changes at the interface are discussed in the DFT section. Figure 1b shows the XRD patterns of thermally treated samples. At 600 °C, the presence of distinctive peaks is indicative of the CsTO phase remaining unaltered. During the thermal treatment, the existing C_16_TMA^+^ decomposes, and the remaining structure is solely inorganic, partially Cs-vacant, and partially exfoliated CsTO. In Figure 1b, the additional impact of varying temperatures is elucidated by XRD patterns. At all temperatures, peaks attributed to the unreacted C_16_TMA have disappeared. The diffraction peaks at 600 °C of treatment have not changed; however, at 700 and 800 °C, the peaks have disappeared, confirming that the layers recrystallize and lose the symmetric pattern of stacking (i.e., long-range order). The peaks are still sharp in the case of (C_16_TMA)_0.25_CsTO-600, which can be ascribed to the synthesis temperature of bulk CsTO, which is 600 °C. This transformation contributes to changes in the interlayer spacing as carbonaceous layers become more prevalent with higher treatment temperatures.

In the Raman spectroscopy measurement, exf-CsTO-700 reveals sharper peaks (Figure 1c,d) than all samples, corroborating that although the XRD patterns are diminishing in the case of exf-CsTO-700, there could be a string crystallinity that these sharp peaks have appeared. Interestingly, increasing the temperature to more than 700 °C (exf-CsTO-800) causes a detrimental effect on the intensities of the peaks. Furthermore, a new distinct peak at 143 cm^–1^ appears in heat-treated samples, compared to the pristine CsTO, is attributed to the *E*_1g_ vibrational mode and it only appears after exfoliation and heat treatment. This same peak is also present in the heat-treated samples of (exf-CsTO). This observation implies that the optimal temperature to get a highly crystalline structure is 700 °C.

The UV–vis spectra obtained by the diffuse reflectance technique were extrapolated into Tauc plots through Kubelka–Munk transformation for band gap energy determination (Figure 2a,b).^53^ It is experimentally observed that bulk CsTO has the largest energy gap (*E*_g_ = 3.64 eV)^53^ among all thermally treated exf-CsTO samples.^54^ Possessing such a wide band gap can be one of the main reasons for exhibiting limited photocatalytic activity under solar light irradiation.^55^ Accordingly, the band gap of exf-CsTO-700 (*E*_g_ = 3.28 eV) is slightly smaller than both exf-CsTO-600 (*E*_g_ = 3.36 eV) and exf-CsTO-600 (*E*_g_ = 3.40 eV). This red shift in the band absorption and subsequent band gap reduction can be feasibly ascribed to the cesium vacancy and the partial exfoliation of CsTO nanosheets. More details on the electronic structure are discussed in the DFT section.

**Figure 2:**
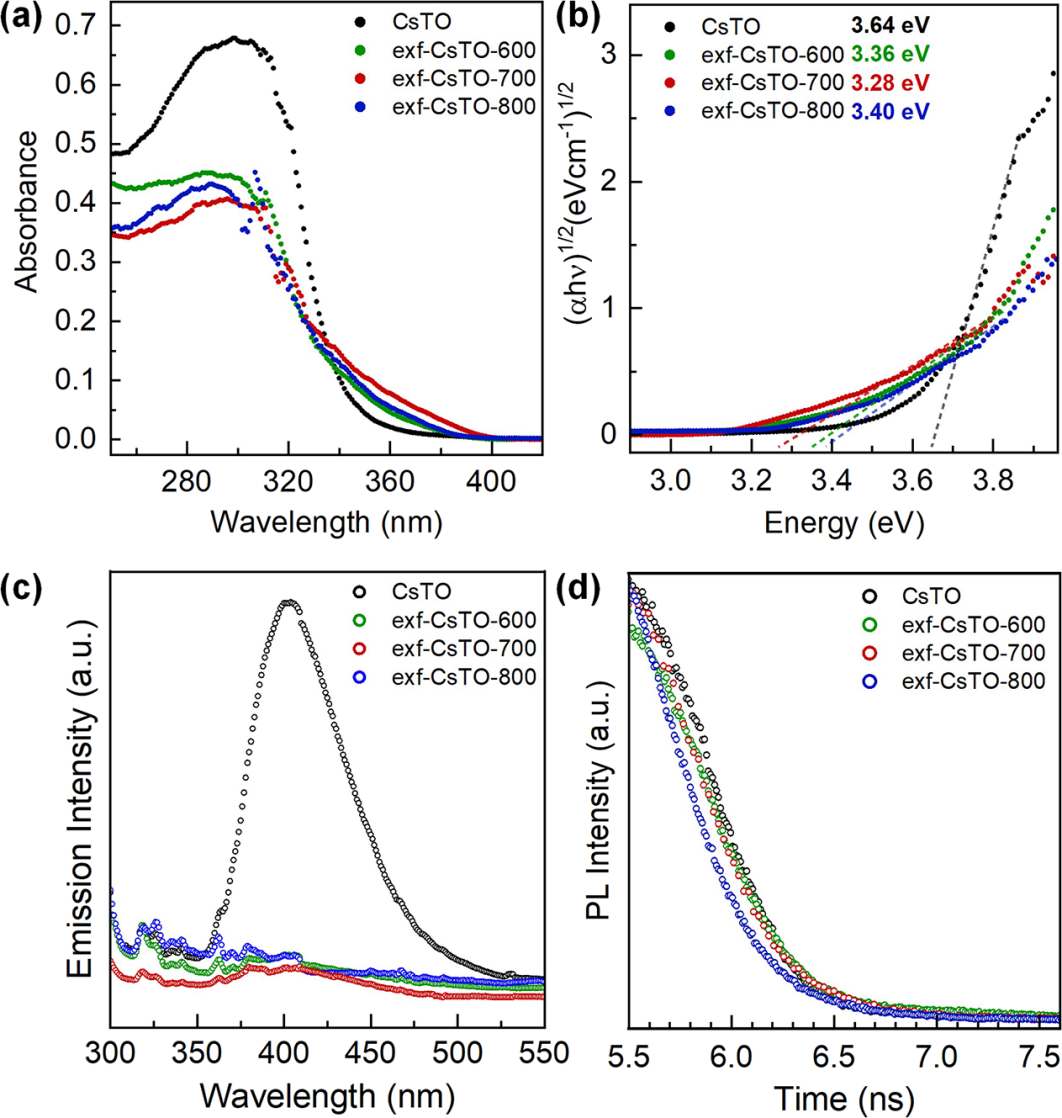
(a) UV–vis spectra of corresponding samples, and (b) their extrapolated Tauc plots derived from Kubelka–Munk conversion. (c) The photoluminescence (PL) emission spectra, and (d) the time-resolved PL decay of the samples.

The red shift in the UV–vis absorption band is the result of the defects that are induced by C_16_TMA^+^ treatment and thermal treatment, corroborating the electronic structure changes brought on by the approach we have applied here. This red shift can also contribute to further enhanced light harvesting through longer wavelengths, especially in exf-CsTO-700, leading to potentially higher PC and PEC activities, compared to the other two thermally treated samples. The fact that anatase TiO_2_ has a wide band gap (*E*_g_ = 3.2 eV), and is mainly functional at UV wavelengths (<400 nm), is widely known.^56^

PL emission spectra of the samples are shown in Figure 2c. Generally, it is believed that the higher PL signal is related to the fast charge carrier recombination.^57^ Emission spectra with a maximum peak at 403 nm were observed for CsTO, showing that the charge carrier recombination is significantly diminished for all thermally treated exf-CsTO. Cs vacancies in these samples are probably the leading cause of the diminishing PL peak intensities. Accordingly, exf-CsTO-700 has the lowest intensity peaks in the emission range of 300–450 nm among other thermally treated samples.^54^ PL lifetime decay profiles of the samples also show a very similar trend for the samples (Figure 2d), which will be discussed further. This result also shows that exf-CsTO-700 not only has a narrower band gap, but the electron–hole recombination has also been significantly blocked in the performance of the semiconductor.

FTIR spectra of the following samples are shown in Figure S2a. Broad infrared bands at 1628 cm^–1^ and 3370–3600 cm^–1^ point to the existence of H_3_O^+^ ions or H_2_O molecules in the interlayer space and/or surface.^58^ Also, *n*-alkylamine peaks are evident within the FTIR spectra of *n*-alkylamine-exchanged CsTO by assigning the −CH_2_ bending at 1470 cm^–1^, as well as sp^3^-hybridized C–H bond stretching vibrations spanning the range of 2855–2955 cm^–1^.^59^ The peak at 721 cm^–1^ is attributed to O–Ti–O bending vibrations and Ti–O stretching of TiO_6_ octahedral groups,^60^ While 910 cm^–1^ is related to the Cs–O bonding.^61^ It is also shown that C_16_TMA^+^ presence with the C–H stretching modes in the range 2800–3000 cm^–1^ are the dominant feature in the FTIR spectra resulting from C_16_TMA^+^. The symmetric and asymmetric CH_2_ stretching modes provide the two distinct peaks at 2921 and 2850 cm^–1^.^62^ After heat-treating (C_16_TMA)_0.25_CsTO (Figure S2a), the characteristic peaks of C_16_TMA^+^ have disappeared, corroborating their complete decomposition (Figure S2b).^63^ The thermal gravimetry analysis of the exf-CsTO-700 also showed insignificant weight loss, confirming that the C_16_TMA^+^ species are totally removed from the heat-treated sample (Figure S3).

XPS measurements were performed to investigate the surface characteristics of the respective samples. Figure 3a displays the Ti 2p spectra, revealing distinct peaks at approximately 458.0 and 464.4 eV for Ti 2p_3/2_ and Ti 2p_1/2_, respectively. Surface oxygen defects can serve as active adsorption sites, which are crucial in preventing the recombination of electron–hole pairs at the surface. This can improve the charge carrier separation, thus increasing photocatalytic activity. In the O 1s spectra (Figure 3b), the primary peak at 530.1 eV is attributed to the lattice Ti–O–Ti oxygen,^64^ while a slight shoulder at approximately 531.0 eV is often associated with the surface hydroxyl groups or surface oxygens.^65^ Figure 3c depicts peaks at around 738.1 and 724.3 eV for Cs 3d_3/2_ and Cs 3d_1/2_, respectively. Notably, both peaks shift slightly toward lower binding energies after heat treatment.

**Figure 3:**
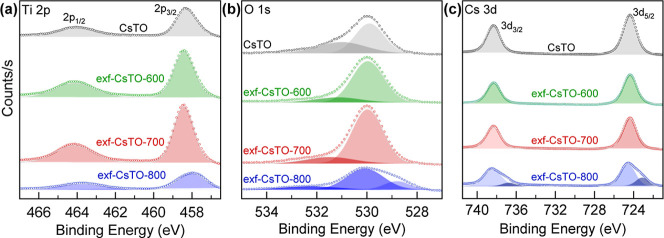
XPS spectra of pristine CsTO with exf-CsTO-*x* samples in Ti 2p (a), O 1s (b), and Cs 3d (c) elements.

This shift suggests changes in the layered titanate’s crystallinity induced by Cs. According to the atomic ratio of Cs in heat-treated samples compared to the bulk CsTO, the Cs atomic percent has significantly decreased (almost one-third of Cs has been removed). The low binding energy values of Ti 2p and Cs 3d XPS spectra of exf-CsTO-800 indicate that the lattice structure likely undergoes significant changes, including defect formation, when exposed to an inert atmosphere at 800 °C. Such a reduction could negatively affect the activity of this sample.

Figure 4a–d represent the SEM images of the bulk CsTO and the modified samples, revealing remarkable changes in the morphology once treated with C_16_TMA^+^ and further heat treatment of CsTO at high temperatures (600–700 °C). To confirm that the disaggregation and exfoliation of CsTO are specifically caused by the combined action of C_16_TMA^+^ and heat, we also heat-treated bulk CsTO at 700 °C (CsTO–700) without prior C_16_TMA^+^ treatment. The SEM results show that the particle sizes and stacking of the layers have grown even larger (Figure S5). Comparing the heat-treated samples also reveals that raising the temperature from 600 to 700 °C does not cause a tangible change in the layer thicknesses, while increasing it from 700 to 800 °C leads to a remarkable growth in the thickness and the stacking of the layers. The bulk CsTO overall exhibits a lateral size of nearly 1 μm and an extreme stacking of the layers. Specifically, C_16_TMA^+^-treated samples, denoted as (C_16_TMA)_0.25_CsTO, show an average lateral size of 400 nm, as exemplified in Figure S4. It is noteworthy that the combined C_16_TMA^+^ and heat treatments do not induce significant disruptions to the layered structure of CsTO while leading to a substantial reduction in lateral dimensions and the thickness of the aggregates.

**Figure 4:**
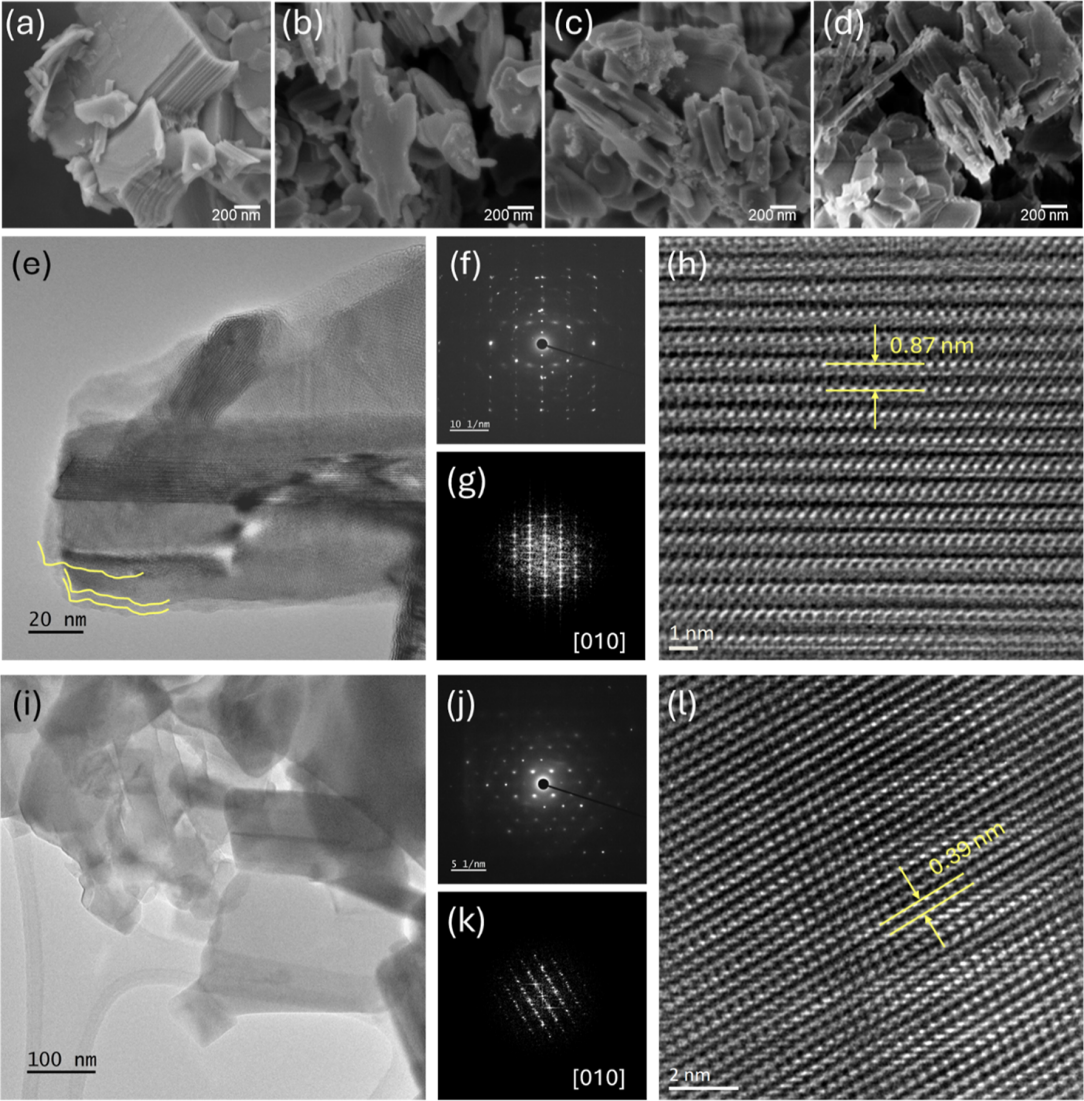
SEM images of (a) pristine CsTO, (b) exf-CsTO-600, (c) exf-CsTO-700, (d) exf-CsTO-800. (e,i) Low-magnification TEM images; (f,j) SAED patterns; (g,k) fast Fourier transform (FFT) images along the [010] zone axis; (h,l) HRTEM images of cation-unexchanged CsTO-700 and exf-CsTO-700, respectively.

The morphology and atomic-scale structural variations between the cation-unexchanged and exfoliated CsTO-700 samples were investigated using HRTEM. As shown in Figure 4e,i, the low-magnification TEM images reveal that the unexchanged sample exhibits a bulk morphology composed of multiple stacked layers. At the same time, the exfoliated CsTO-700 displays a distinct layered structure with well-separated sheets, confirming successful exfoliation. These observations are further supported by the SAED patterns in Figure 4f,j. The cation-unexchanged sample exhibits a polycrystalline diffraction pattern. In contrast, the exfoliated material presents well-defined spots indicative of a predominantly single-crystalline nature, likely a result of the delamination of individual layers during exfoliation. Additionally, fast Fourier transform (FFT) images obtained along the [010] zone axis (Figure 4g,k) demonstrate that the cation-unexchanged sample exhibits a characteristic large interlayer spacing of approximately 0.87 nm, which decreases to 0.39 nm after cation exchange. The TEM-based elemental mapping of a particle captured by the STEM method also shows that Cs (as well as other composing elements of CsTO), after the cation exchange with C_16_TMA^+,^ is still partially left (Figure S6).

## PEC and PC Activity Investigations

In Figure 5a, the recorded LSV measurements reveal the photoelectrochemical activity for the OER with the tested samples. Remarkably, the trends observed in photocurrent align closely with the results obtained from the optical characterizations, consistently signifying that the treatment of bulk CsTO and further calcination at 600 and 700 °C improve its PEC activity. Additionally, in the context of PEC analyses, a carbonaceous layer formed from CTAB may establish an additional conductive layer. This result is in agreement with UV–vis absorption spectroscopy and the band gap calculation from the Tauc plot, as well as the PL lifetime decay results. The photocurrent density of bulk CsTO is notably lower than that of the heat-treated samples (600 and 700 °C). However, the exf-CsTO-800 shows the lowest photocurrent, even compared to the bulk. This observation suggests that thermal treatment at 800 °C leads to a collapse of the PEC potential of the material’s structure. This low photocurrent for exf-CsTO-800 is consistent with the SEM images, showing increased particle sizes at 800 °C, causing the reduction of the active surface area, as further supported by the EIS results (Figure 5b).

**Figure 5:**
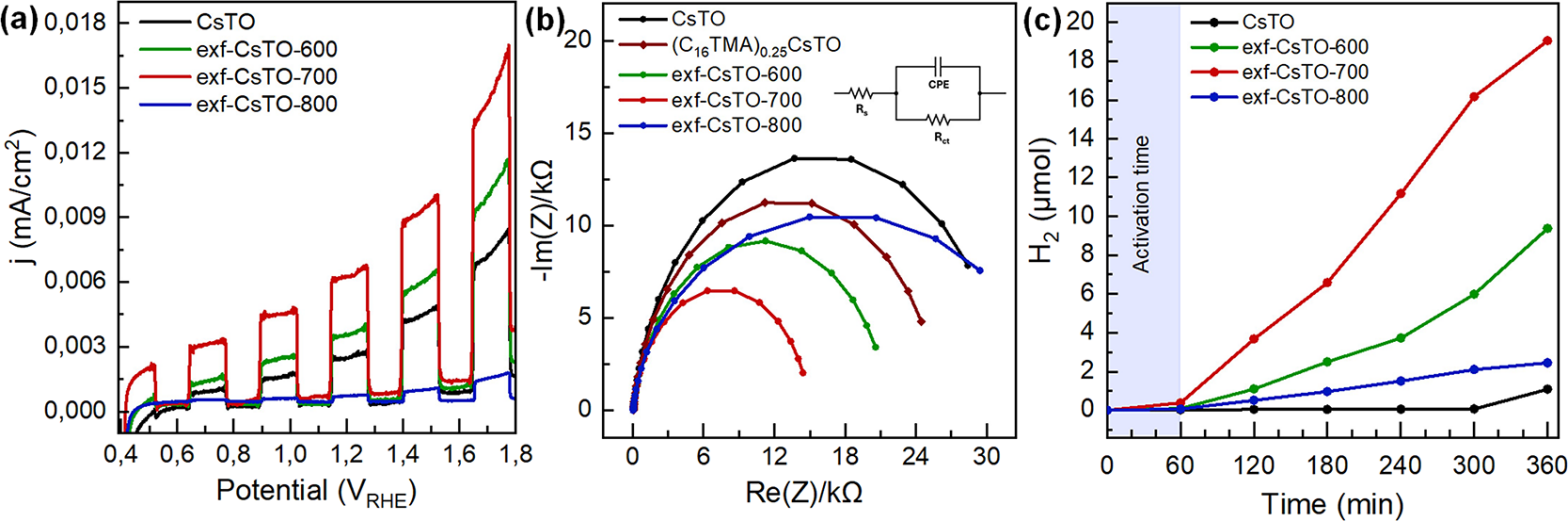
(a) OER activity followed by LSV measurements, (b) EIS measurement of pristine CsTO and C_16_TMA^+^ and after heat treatment at different temperatures at 1.8 *V*_RHE_ in 0.1 M Na_2_SO_4_ at pH = 7, Randle’s model used, as shown in the inset, and (c) PC activity comparison of corresponding samples for HER by water splitting of CsTO and C_16_TMA^+^-treated samples and after heat-treatment at different temperatures.

EIS measurements were performed to reveal the capacitive behavior and charge carrier conductivity of the samples through plotting the imaginary resistance versus the real resistance, which is known as the Nyquist plot, Figure 5b. The fitting was done by using Randle’s model with a constant phase element (CPE), and it is shown in Figure 5b as an inset. *R*_s_ is the resistance of the electrolyte; the CPE corresponds to the capacitance between the electrolyte and the electrode and was used instead of an ideal capacitor in this model to account for surface inhomogeneity, electrode porosity, and surface roughness of the prepared photoelectrodes, and *R*_ct_ is the charge transfer resistance.

The EIS results show almost the similar trend as the LSV measurement results. While bulk CsTO shows *R*_ct_ = 138.3 kΩ, the heat treatment at 600 °C, 700 °C, and 800 °C reduces the *R*_ct_ further to 65.8 kΩ, 59.2 kΩ, and 34.2 kΩ, respectively. The outcomes of the EIS closely match the PEC response, where exf-CsTO-700 has higher catalytic activity. The lower *R*_ct_ of exf-CsTO-700 can be one of the reasons why it shows more promise for photoelectrocatalysis.

The observed effect of temperature on the PEC measurements is similar to the trends observed in the PC results, reinforcing the influence of temperature on the overall catalytic behavior. In Figure 5c, it is evident that the bulk CsTO exhibits weaker PC activity in the HER. Furthermore, the PC activities of the exf-CsTO-*x* are significant, resulting in hydrogen production levels of 19.1 μmoles for exf-CsTO-700 samples (Figure 5c). On the other hand, PL lifetime decay shows that the recombination of charge carriers is the slowest for exf-CsTO-600 and exf-CSTO-700 samples, which can explain the best activity observed. In Figure S7, it is also presented that the PEC activity increase is not merely dependent on the C_16_TMA^+^ treatment with CsTO (C_16_TMA)_0.25_-(CsTO) since the (C_16_TMA)_0.25_-CsTO sample showed a lower activity than its corresponding heat-treated version (exf-CsTO-700).

The recombination of the charge carriers appears to be slowest for exf-CsTO-600 and exf-CsTO-700 samples, which reflects on the photo­(electro)­catalytic activities. In Figure 5a,c, exf-CsTO-700 exhibits the highest level of activity. This result agrees well with UV–vis absorption spectroscopy and PL lifetime decay results. The relatively low photocurrent density shown by the bulk CsTO can be attributed to the property of its as-prepared layered structure. The bulk pristine CsTO has limitations in recieving the incident light intensity and available surface area for catalytic reactions. Furthermore, it is plausible that the CsTO layers may undergo agglomeration on the electrode surface, further diminishing the effective surface area for the reaction.

## Density Functional Theory Calculations

Ab initio DFT calculations were carried out to model the interfacial structure at the atomic level and to understand the electronic structure of CsTO. The initial structural model consisted of an orthorhombic lepidocrocite-type stoichiometric TiO_2_ bilayer system containing a total of 24 atoms.^66^ The structure exhibits a two-dimensional configuration with distinct interlayer regions available for atomic intercalation. These interlayer spaces were systematically modified through the incorporation of Cs atoms and H substitutions. The compositional variations were systematically explored through configurations with complete Cs intercalation (4 × Cs atoms), and partial H substitution, with compositions ranging from Cs_4–*x*_H_*x*_ (*x* = 0 to 4), as depicted in Figure 6.

**Figure 6:**
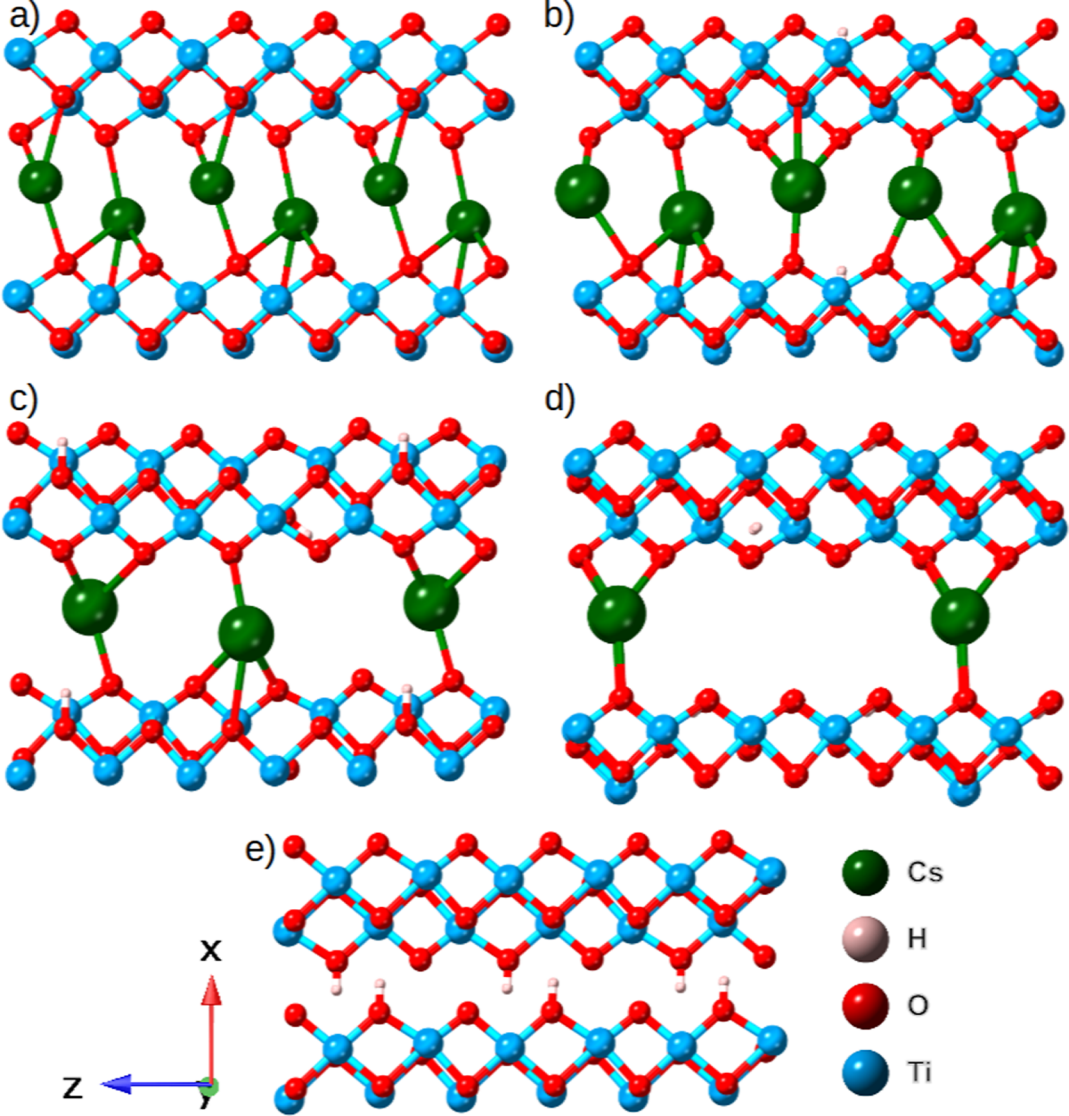
Structural evolution of TiO_2_ as a function of the Cs/H concentration. The top-left (a) image represents the bare TiO_2_ bilayer. The ratio between H and Cs varies from a fully hydrogen- to a fully Cs-intercalated structure with 4 × Cs atoms in each unit cell (b–e). The front-to-back in-plane and the out-of-plane directions have been expanded by a factor of 2 to enhance visual clarity and facilitate detailed structural interpretation.

The evolution of the interplanar distance—which considers the thickness of a single TiO_2_-based layer plus the interfacial (d-basal) space—throughout the Cs doping process is presented in Figure 7. The DFT-relaxed structure shows that while the inclusion of the first Cs atom increases the *d*-space by 3.4 Å compared to the bare TiO_2_, this enlargement is moderated by up to 0.9 Å upon Cs addition, due to the formation of stable Cs–O bonds at the interface. The inclusion of H in the interface is small, and the difference between the bare bilayer and the fully H-populated one is within the error bar of our estimations.

**Figure 7:**
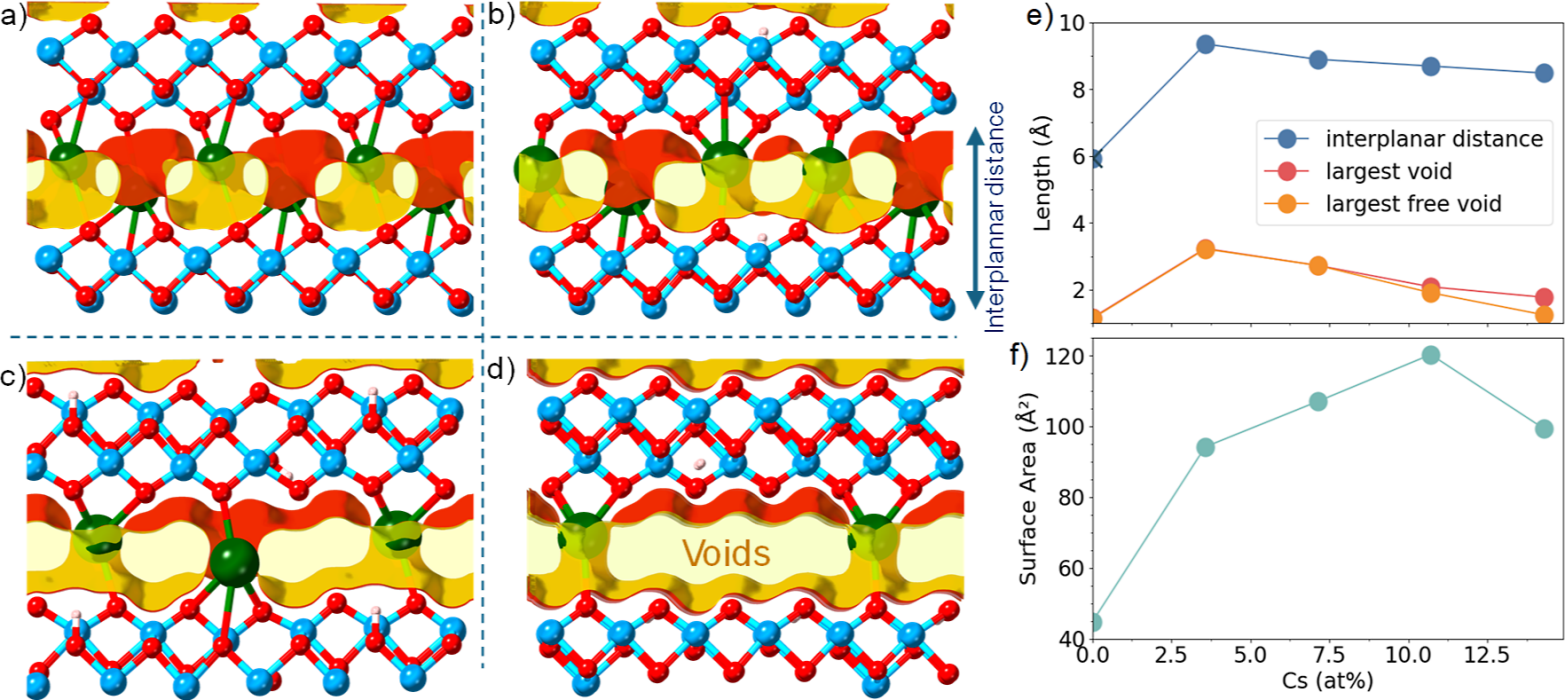
Representation of the interplanar space as a function of the Cs concentration and with respect to the 28-atom composition Ti_8_O_16_Cs_4–*x*_H_*x*_ for *x* = 0 (a) to 4 (b–d). And (e) evolution of the interplanar distance as a function of the Cs concentration and (f) surface area values with respect to the pristine TiO_2_ model. The points in the graphs represent the length of the DFT equilibrium simulation box. The corresponding value for the TiO_2_ bilayer length (0% Cs) is marked with ×.

Voronoi network analyses are used to quantify the void space in the porous material. The calculations give us the diameter of the largest spherical volume that could fit in the interfacial space and the one that could travel through it. Figure 7 shows that the composition with the lowest Cs concentration holds the largest included sphere, which is also able to travel through the interface. This can be understood by a simple visual inspection of the structure (Figure 6), which has the largest interlayer space, with a minimal space occupation by the large Cs atoms. As the Cs concentration increases, the diameter of the largest accommodated sphere is reduced. For more than 10% of Cs, this cannot travel anymore through the interface and the free volume decreases.

The surface area is integrated by Monte Carlo sampling, using a 0.2 Å diameter spherical probe, and subtracting the value obtained for the bare TiO_2_ model. As expected, the surface area increases to values over 94 Å^2^ for all compositions with Cs, with a maximum of 120 Å^2^ for 10.7 at% Cs concentration.

The partial density of states (PDOS) analyses presented in Figure 8 show the electronic structure of different compositions. The PDOS shows that the states above the Fermi level (*E*_F_) are predominantly composed of Ti 3d orbitals, while O 2*p* contributes strongly to the top edge of the valence band. This behavior is typical in other stable TiO_2_ polymorphs.^67,68^ The energy gaps around 3.3 eV, obtained from hybrid DFT calculations, are in line with our experimental measurements and correct the typical underestimation of standard DFT.

**Figure 8:**
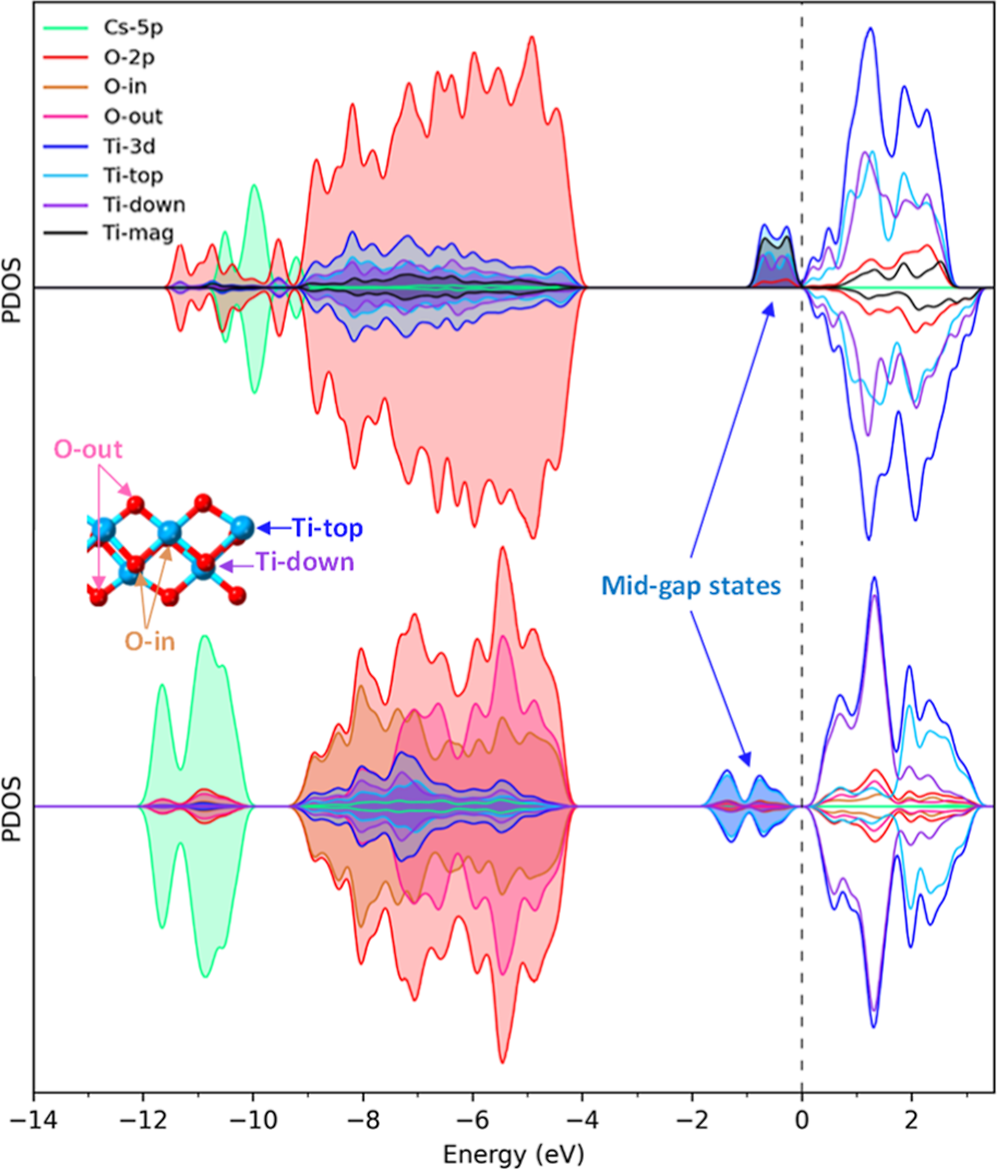
Partial density of states (PDOS) for the lowest and highest Cs-doped compositions (Ti_8_O_16_Cs_4–*x*_H_*x*_ for *x* = 1 and 4). The Fermi level (*E*_F_) is set at zero energy and indicated in the graph with dashed vertical lines. The states over and under the axis represent the spin-up and spin-down components for each composition, respectively. The partial contributions follow the same color code as the structure figures.

The Cs species do not exhibit significant contributions around the *E*_F_, with their states residing at deeper energy levels, lying at the bottom of the region occupied by O. Interestingly, the presence of Cs at the interface appears to promote the creation of midgap states. According to the existing literature, which discusses the formation and effect of midgap states coming from defects in semiconducting metal oxides, such as TiO_2_,^69–72^ HfO_2_ ^73^ or ZnO^74,75^ and other materials such as PbSe.^76^ These midgap states can affect the catalytic activity by providing: (i) accessible energy levels within the band gap, extending activity to the visible light region, (ii) effective trapping centers for photogenerated charge carriers (electrons or holes), inhibiting their recombination and increasing lifetime for charge carriers available for redox reactions, and (iii) direct active sites for surface reactions. In our case, these states are mainly associated with Ti atoms that have nonzero magnetic moments. For example, at low Cs compositions, magnetized Ti species are responsible for the spin-up states at the bottom of the conduction band, which form a pseudogap around E_F_, which reduces the energy gap in the spin-up component. For higher Cs compositions, the magnetization shows a layered distribution, exhibiting one line of Ti atoms with zero and the other with a nonzero magnetic moment within the same TiO_2_ layer. The O contribution to the PDOS has also been divided between outer O atoms, which can bond to Cs, and inner O, which form bonds only with Ti atoms. In this case, the midgap states are more stable, situated further from *E*_F_, and they are present in all Ti atoms species. Midgap states, often introduced by defects such as vacancies or dopants, can extend light absorption to the visible region and serve as localized sites facilitating charge transfer between the semiconductor and adsorbed species, eventually promoting catalytic reactions. In our case, we observe that an optimally controlled Cs doping produces midgap states that can act as trapping sites for charge carriers, potentially enhancing the catalytic activity of the doped-TiO_2_ system.^68,77^ Nevertheless, we should also mention that these states can act as efficient traps for photogenerated charge carriers, enhancing recombination, which ultimately reduces carrier lifetimes and hampers charge transport. Thus, while an excess of such defect sites can be detrimental, the strategic incorporation of midgap states via controlled doping and precise band gap engineering can be used to significantly enhance the photocatalytic activity of CsTO.^69,78^

## Conclusion

In conclusion, our strategy—combining partial cation exchange of Cs with C_16_TMA^+^ and subsequent high-temperature calcination—was key for enhancing the PEC and PC activities of layered cesium titanate. By leveraging the large atomic radius and low exchangeability of Cs, we were able to create significant interlayer voids and atomic defects through the controlled partial removal and replacement of Cs ions. The Cs-vacant CsTO simultaneously revealed enhanced catalytic activity after recrystallization and calcination at high temperature were performed. This approach created atomic voids, higher surface area, midgap, and narrower band gap, which lowered the charge carrier resistance, prolonging the photogenerated electron/hole, and eventually, enhancing the PC and PEC activities. It seems that due to the microsized lateral size range of the CsTO, full exfoliation may not be feasible in this material. The lateral dimensions of CsTO impeded the effective intercalation of C_16_TMA^+^ into its interlayer spaces, a factor that likely led to the partial creation of Cs cation vacancies. The varying activities observed at different temperatures are primarily linked to the crystallization and subsequent decomposition temperatures of the carbonized C_16_TMA^+^, being 700 °C the optimum thermal treatment temperature. The LSV measurements indicate that the material holds significant promise as a dual-functional material, acting as a photoelectrocatalyst for OER and a photocatalyst for HER.

## Supplementary Material



## Notes

The DFT data sets are available in the NOMAD repository under the following DOI: https://nomad-lab.eu/prod/v1/gui/dataset/doi/10.17172/NOMAD/2025.08.22-1; Figure S1: XRD patterns of C_16_TMABr, (C_16_TMA)_0.25_CsTO, bulk CsTO, and the PDF reference XRD patterns of Cs_2_Ti_6_O_13_ and TiO_2_; Table S1: The crystal parameters of CsTO, obtained from the XRD pattern and the theoretical calculations; Figure S2: (a) FTIR spectra of CsTO, (C_16_TMA)_0.25_CsTO, and (b) exf-CsTO-x samples; Figure S3: Thermal gravimetry analysis (TGA) of the samples; Figure S4: SEM image of the (C_16_TMA)_0.25_CsTO; Figure S5: SEM images of cation-unexchanged CsTO-700; Figure S6: (a) STEM image of exf-CsTO-700 and its TEM-based elemental mapping graphs of (b) Cs K_α1_, (c) O K_α1_, (d) Ti K_α1_ of exf-CsTO-700 captured from the same particle; Figure S7: LSV curves of the samples under chopped light illumination in 0.1 M Na_2_SO_4_ with a scan rate of 10 mVsec^–1^ (PDF)
