# Supplementary material for: Interlayer Atomic Voids by Partial Cesium Defect in Layered Titanate Activate Photo(electro)catalytic H2 and O2 Generation
Source: ACS Appl Energy Mater. 2025 Sep 11;8(20):15079–91. doi: 10.1021/acsaem.5c01514 (PMC12606251; doi:10.1021/acsaem.5c01514)
Supplement: Supplementary file 1 [file ae5c01514_si_001.pdf]

Supplementary Information

Interlayer atomic voids by partial cesium defect in layered titanate activate photo(electro)catalytic H_2 and O_2 generation

Tuğçe Üstünel,[1,2] José Julio Gutiérrez Moreno,[3] Xiaoran Zheng,[4] Sajjad S. Mofarah,[4] Hadi Jahangiri,[5] Sarp Kaya,[*,2,6] Esmail Doustkhah[*,7,8]

[1] *Materials Science and Engineering, Koç University, 34450 Istanbul, Türkiye*

[2] *Koç University Hydrogen Technologies Center (KUHyTech), 34450 Istanbul, Türkiye*

[3] *Barcelona Supercomputing Center, Plaça d'Eusebi Güell, 1-3, 08034 Barcelona, Spain*

[4] *School of Materials Science and Engineering, UNSW Sydney, Sydney, NSW, 2052 Australia*

[5]*Koç University Surface Science and Technology Center (KUYTAM), Koç University, Sariyer 34450, Istanbul, Türkiye*

[6]*Department of Chemistry, Koç University, 34450 Istanbul, Türkiye*

[7] *Chemistry Department, Faculty of Engineering and Natural Sciences, Istinye University, 34396 Sariyer, Istanbul, Türkiye*

[8] *Clean Energy Research Center (TEAM), Istinye University, 34396 Sariyer, Istanbul, Türkiye*

* Corresponding authors: sarpkaya@ku.edu.tr (Sarp Kaya), esmail.doustkhah@istinye.edu.tr (Esmail Doustkhah)

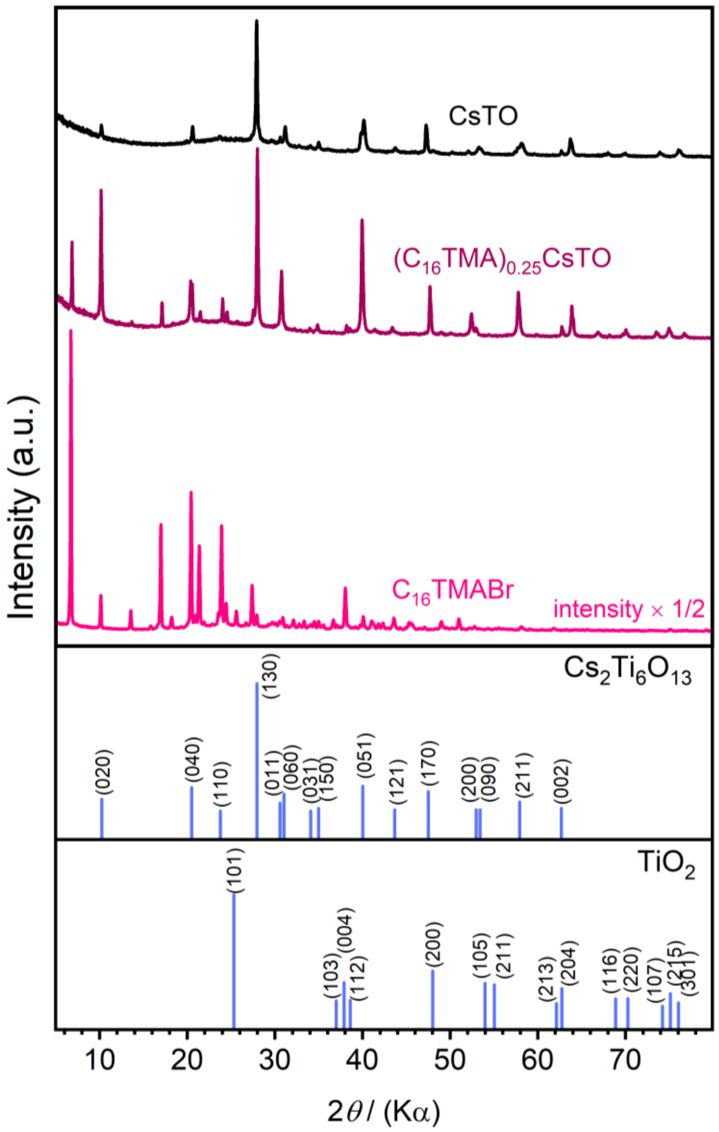

Figure S1. XRD patterns of $C_{16}TMABr$, $(C_{16}TMA)_{0.25}CsTO$, bulk CsTO, and the PDF reference XRD patterns of $Cs_2Ti_6O_{13}$ and TiO_2.

The lattice parameter calculation was performed by the following formula of the orthorhombic crystal structure: $\frac{1}{d^2} = \frac{h^2}{a^2} + \frac{k^2}{b^2} + \frac{l^2}{c^2}$, where h, k, l indicate the Miller indices (h, k, l) and a, b, c indicate the lattice parameters. The lattice parameters were obtained by placing the following values (Table S1) in the formula and calculating the lattice parameters accordingly:

Table S1. The crystal parameters of CsTO, obtained from the XRD pattern and the theoretical calculations.

2θ (°)	(hkl)	d_{exp} (Å)	d_{calc} (Å)	Error (%)
10.18	(0, 2, 0)	8.6853	8.6935	0.09

20.64	(0, 4, 0)	4.3037	4.3066	0.07
31.18	(0, 6, 0)	2.8682	2.8688	0.02
40.04	(0, 5, 1)	2.2511	2.2530	0.09
47.22	(1, 7, 0)	1.9233	1.9260	0.14

Based on the above information obtained from the XRD pattern of the Bulk CsTO, the lattice parameters are a = 4.6341 Å, b = 11.2156 Å, c = 5.8178 Å. Since the error% is less than 0.15%, the obtained values are reliable.

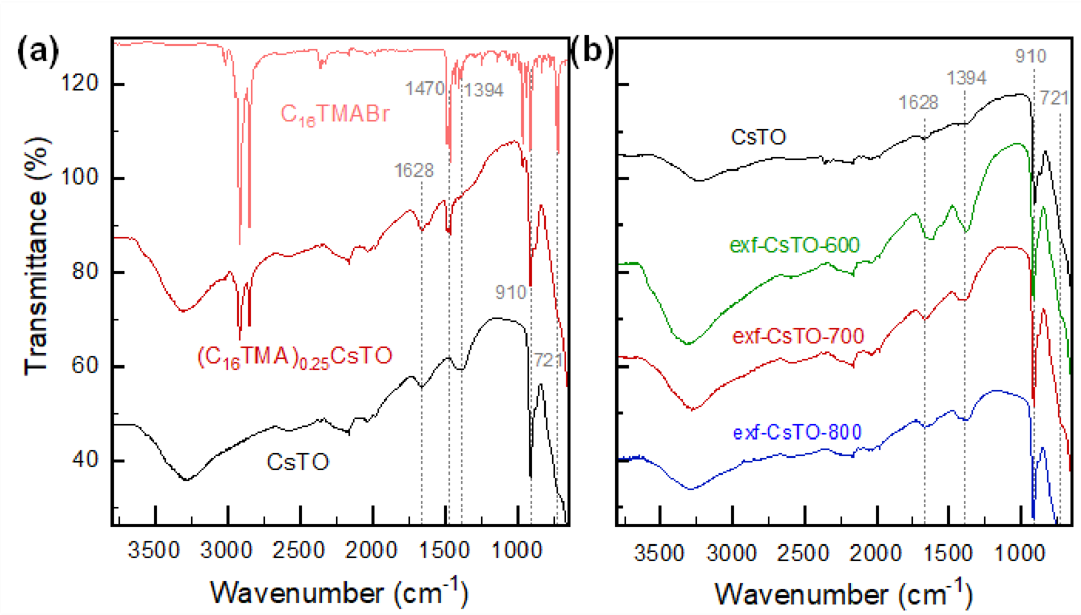

Figure S2. (a) FTIR spectra of CsTO, $(C_{16}TMA)_{0.25}CsTO$, and (b) exf-CsTO-x samples.

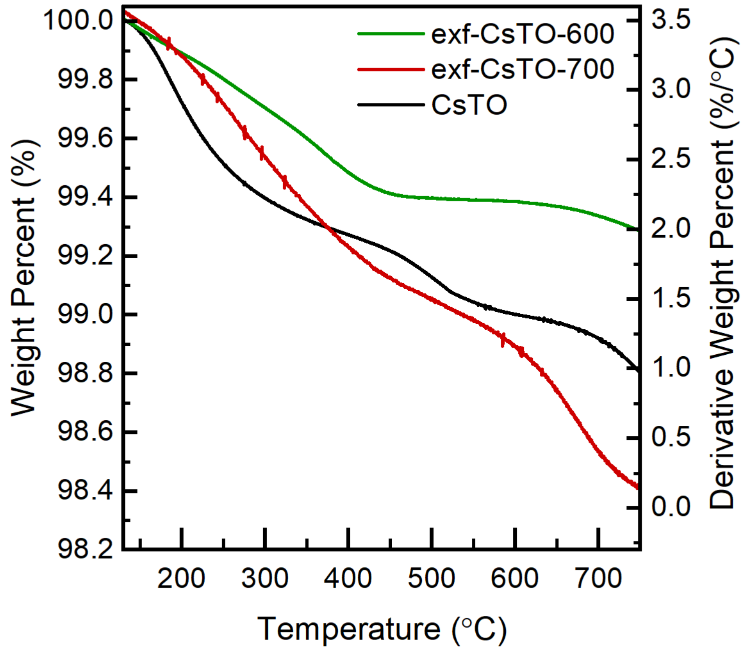

Figure S3. Thermal gravimetry analysis (TGA) of the samples.

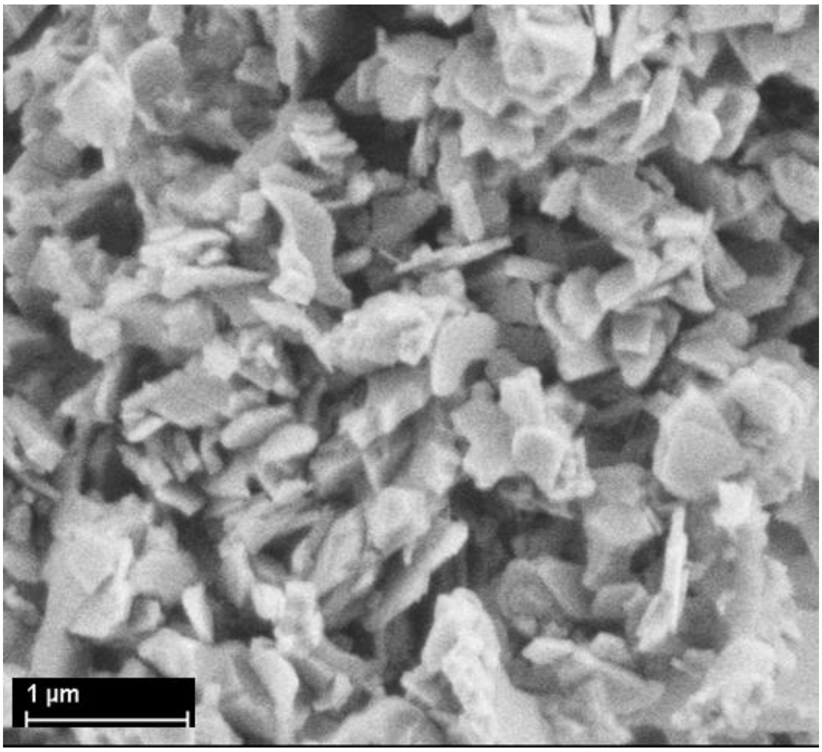

Figure S4. SEM image of the $(C_{16}TMA)_{0.25}CsTO$.

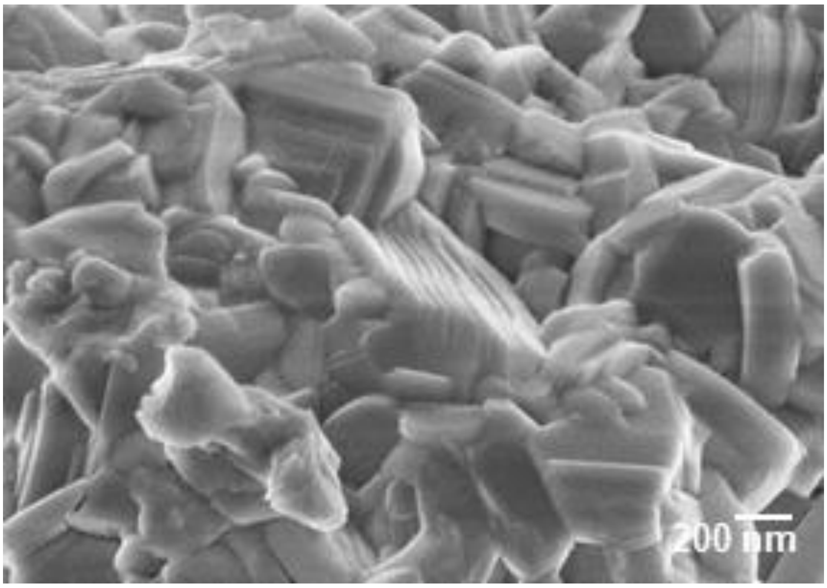

Figure S5. SEM images of cation-unexchanged CsTO-700.

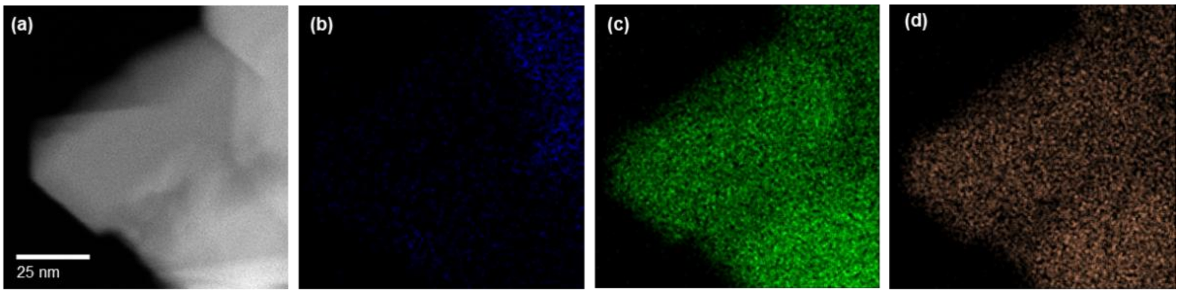

Figure S6. (a) STEM image of exf-CsTO-700 and its TEM-based elemental mapping graphs of (b) Cs $K_{\alpha1}$, (c) O $K_{\alpha1}$, (d) Ti $K_{\alpha1}$ of exf-CsTO-700 captured from the same particle.

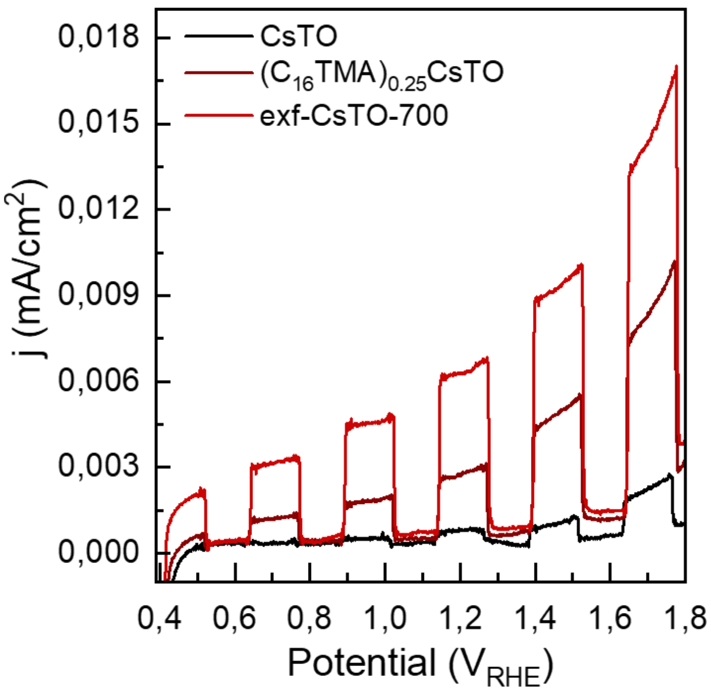

Figure S7. LSV curves of the samples under chopped light illumination in 0.1 M Na_2SO_4 with a scan rate of 10 mVsec^{-1}.
